# Tamoxifen affects chronic pancreatitis‐related fibrogenesis in an experimental mouse model: an effect beyond Cre recombination

**DOI:** 10.1002/2211-5463.12714

**Published:** 2019-09-07

**Authors:** Xuan Li, Christian Clappier, Ingo Kleiter, Rainer Heuchel

**Affiliations:** ^1^ Pancreas Cancer Research (PaCaRes) Lab Department of Clinical Science, Intervention and Technology Karolinska Institutet Stockholm Sweden; ^2^ Department of Neurology Ruhr‐Universität Bochum Germany

**Keywords:** Cre‐LoxP, fibrosis, pancreatitis, tamoxifen

## Abstract

Tamoxifen is very successfully used for the induction of Cre^ERT^‐mediated genomic recombination in conditional mouse models. Recent studies, however, indicated that tamoxifen might also affect the fibrotic response in several disease models following administration, both *in vitro* and *in vivo*. In order to investigate a possible effect of tamoxifen on pancreatic fibrogenesis and to evaluate an optimal treatment scheme in an experimental pancreatitis mouse model, we administered tamoxifen by oral gavage to both male and female C57BL/6J mice and then waited for different periods of time before inducing chronic pancreatitis by cerulein. We observed a sex‐specific and time‐dependent effect of tamoxifen on the fibrotic response as measured by collagen deposition and the number of myofibroblasts and macrophages. The findings of *in vitro* studies, in which cerulein was administrated with or without 4‐hydroxytamoxifen to stimulate primary murine female and male pancreatic stellate cells, supported our *in vivo* observations. Real‐time PCR also indicated that this effect may be related to differences in ERα expression between female and male stellate cells. Our data demonstrate that tamoxifen administration has unignorable side effects, which affect the experimental outcome in a cerulein‐based model of chronic pancreatitis in mice. We suggest a 2‐week waiting period before cerulein administration to reduce side effects to a minimum for the described fibrosis model in female mice.

**Abbreviations**
CI: confidence interval
DCs: dendritic cells
DMEM/F12: Dulbecco's modified Eagle's medium/F12 medium
ECM: extracellular matrix
ER: estrogen receptor
PSCs: pancreatic stellate cells
PS: penicillin/streptomycin
SERM: selective estrogen receptor modulator

The Cre‐LoxP system is widely used in genetically engineered mice. Usually, Cre is under the control of a promoter that restricts its expression to a particular tissue or cell type, resulting in the deletion or inversion of genomic DNA sequences that are flanked by two LoxP sites 1. In addition to tissue‐specific recombination, there is an exogenously inducible Cre‐LoxP variant, which allows turning on the Cre activity, not only in a cell type‐/tissue‐specific fashion, but also at a desired time point. This is managed by utilizing a mutated form of the ligand‐binding domain of the estrogen receptor (ER), fused to the Cre recombinase, forming Cre^ERT^. Cre^ERT^ is only activated and translocated into the nucleus upon binding of active tamoxifen 1, 2.

This system has been broadly utilized to generate stage/tissue‐specific knockouts/knockins in many different cell types/tissues due to its low leakiness and robust Cre activity following tamoxifen treatment. Required dosage and multiplicity of tamoxifen administration, however, depend on multiple factors, such as the route of administration and target tissue 3. The most common treatment regimens are intraperitoneal injections, oral gavage, or topical application (skin) of tamoxifen on one or several consecutive days. This is followed by varied drug‐free days after the last tamoxifen application in order to allow for optimal recombination before further interventions 4, 5, 6. However, recent studies indicated that tamoxifen not only exerts the role of activating Cre recombinase, but also has various side effects in different genetically engineered mouse models 4, 5, 7.

Chronic pancreatitis is commonly defined as a continuing, chronic, inflammatory process of the pancreas, characterized by irreversible fibrosis 8. One of the most frequently used animal models in pancreatitis research is the cerulein‐induced chronic pancreatitis in mice 9. Repeated hyperstimulation of the pancreas with the cholecystokinin analog cerulein leads to excessive zymogen activation, destruction of pancreatic tissue, inflammation evidenced by macrophage infiltration, and finally a fibrotic response characterized by excessive collagen deposition through activated pancreatic stellate cells (PSCs)/myofibroblasts 10.

In order to elucidate the influence of tamoxifen on cerulein‐induced pancreatic fibrogenesis and to optimize tamoxifen administration for an inducible Cre‐LoxP system, we induced experimental chronic pancreatitis in both male and female mice following different tamoxifen ‘fading‐out periods’ and compared the fibrotic response/pathogenesis among those groups.

## Materials and methods

### Animal experiments

C57BL/6J mice between 2 and 3 months of age were treated with tamoxifen oral gavage once per day for five consecutive days. Three days, 1, 2, or 3 weeks after the last oral gavage, mice were subjected to chronic pancreatitis by 2 weeks of cerulein injection, respectively. In the cerulein control group, mice were administrated with corn oil instead of tamoxifen, followed by 2 weeks of cerulein injection 3 days after the last corn oil treatment. In the saline control groups, mice were pretreated with tamoxifen for 5 days, followed by 2 weeks of saline intraperitoneal injection. These control groups were indistinguishable to mice, which received only 2 weeks of saline injection (data not shown). Mice were sacrificed on the third day after the last cerulein injection. Totally, 10 mice were treated per group consisting of five female and five male mice. *Col1a2‐Cre* ^*ERT*^ *‐tdTomato* mice between 2 and 3 months of age were used to isolate male and female PSCs (see below). Tamoxifen oral gavage was given once per day for five consecutive days to activate Cre recombinase according to the protocol described before 11. Stellate cell isolation was performed 1 week after the last tamoxifen treatment. *Col1a2‐Cre* ^*ERT*^ *‐tdTomato* mice were generated by mating B6.Cg‐*Tg(Col1a2‐cre/ERT,‐ALPP)7Cpd/J* mice 12 with B6.Cg‐*Gt(ROSA)26Sor* ^*tm9(CAG‐tdTomato)Hze*^/J mice 13 on a C57BL/6J background.

Cerulein was used to induce chronic pancreatitis as follows: 50 μg·kg^−1^ cerulein (Sigma C9026, Saint Louis, MO, USA) dissolved in saline was injected intraperitoneally every hour, six times per day, 2 days per week for 2 weeks in total 14. Tamoxifen (200 mg·kg^−1^; Sigma T5648) dissolved in corn oil (Sigma C8267) was given by oral gavage once per day for five consecutive days 11. All animal experiments were performed with approval of the local animal ethics committee (Stockholm Södra djurförsöksetiska nämnd: S63‐14; Linköping djurförsöksetiska nämnd: ID 2‐17, ID 91‐15).

### Histological analyses of pancreatitis

Immunofluorescence was performed on pancreatic tissue cryosections (6 μm). Sections were fixed with acetone/methanol (1 : 1) for 15 min at −20 °C. The primary antibodies used were as follows: collagen I (1 : 200, Abcam 292, Cambridge, UK), collagen IV (1 : 200, ab6586), fibronectin (1 : 200, ab2413), vimentin (1 : 100, Cell Signaling 5741, Danvers, MA, USA), CD11b (1 : 50, BD Pharmingen 550282, Franklin Lakes, NJ, USA), and F4/80 (1 : 200, Bio‐Rad, Hercules, CA, USA, CI:A3‐1). Nonspecific binding sites and endogenous biotin were blocked with goat serum (1 : 10, Dako ×0907, Santa Clara, CA, USA) and avidin/biotin blocking kit (Vector Laboratories SP‐2001, Burlingame, CA, USA), respectively. The tissues were incubated with primary antibodies overnight at 4 °C and subsequently with a secondary antibody (goat anti‐rabbit Alexa Fluor 488, 1 : 500, Invitrogen A‐11034, Carlsbad, CA, USA; goat anti‐rat Alexa Fluor 488, 1 : 500, Invitrogen A‐11006) for 1 h at room temperature. Nuclei were counterstained with DAPI. H&E staining and picrosirius red (HistoLab, Västra Frölunda, Sweden, Cat. No. HL27150.0500)/fast green counterstaining (Certistain®, Merck, Kenilworth, NJ, USA, Cat. No. 1.04022) staining were performed on paraformaldehyde‐fixed, paraffin‐embedded pancreatic tissue sections (4 μm). Images were captured using a Zeiss (Oberkochen, Germany) epifluorescence inverted microscope (Axiophot).

Five fields per section were selected randomly at 200× magnification, and five sections per group were analyzed. Morphometric analyses of collagen I, collagen IV, fibronectin, vimentin, CD11b, F4/80, and fibrotic index were performed by using imagej software (National Institutes of Health, Bethesda, MD, USA)^15^. The relative extent of extracellular matrix (ECM) accumulation and mesenchymal cell and inflammatory cell occurrence was determined by the percentage of positive (green fluorescence) staining in the total pancreatic tissue area within the section. The fibrotic index from picrosirius red/fast green staining was calculated as the percentage of collagen area in the total tissue area.

### Cell culture

In order to receive pure PSCs populations for sex‐specific *in vitro* studies, male and female *Col1a2‐Cre* ^*ERT*^ *‐tdTomato* mice (*n* = 3 + 3; C57BL/6J background) were sacrificed and pancreata were digested with collagenase as described before 16. Briefly, pancreatic tissue was cut with scissors into small pieces and digested by collagenase, and cell suspension was washed by HBSS and filtered for FACS. Red fluorescent pancreatic fibroblasts were sorted by FACS based on PE channel (excitation with 496 nm). The primary cells were grown in Dulbecco's modified Eagle's medium/F12 medium (DMEM/F12) supplemented with 10% FBS and 0.5% penicillin/streptomycin (PS) antibiotics. At passages 5–6, 1.6 × 10^5^ cells/well were seeded in 6‐well plates and then cultured with 0.5% FBS + 0.5% PS‐supplemented DMEM/F12 medium for starvation overnight. Afterward, cells were treated with cerulein (final 10^−8^ m) and 4‐hydroxytamoxifen (final 5 × 10^−6^ m; Sigma‐H7904; dissolved in 95% EtOH according to the data sheet and as 1 : 2000 dilution in the final growth medium) as described in the Results section for 24 and 48 h. In the control group, cells received cerulein plus EtOH (1 μL·2 mL^−1^ growth medium).

### PCR

RNA was extracted from cells using the Qiagen (Hilden, Germany) RNeasy kit (74104), and iScript™ cDNA synthesis kit (Bio‐Rad, 1708891) for cDNA preparation. Quantitative real‐time PCR was performed by using SYBR Green kit (Thermo Fisher Scientific, Waltham, MA, USA, K0243) and the Bio‐Rad CFX96 thermal cycler. The following primers were used: *Col1a1* (fwd: GCCAAGAAGACATCCCTGAAG, rev: TGTGGCAGATACAGATCAAGC), *Ctgf* (fwd: AACCGCAAGATCGGAGTGT, rev: TGTGTCTTCCAGTCGGTAGG), *Tgfb1* (fwd: GGCCAGATCCTGTCCAAACT, rev: GCACTGCTTCCCGAATGT), *Serpine1* (fwd: CCGATGGGCTCGAGTATG, rev: TTGTCTGATGAGTTCAGCATC), and *Esr1* (fwd: CTGTCCAGCAGTAACGAGAAAG, rev: CACAGTAGCGAGTCTCCTTGG). Ribosomal protein L13a (*RPL13a*, fwd: GAGGTCGGGTGGAAGTACCA, rev: TGCATCTTGGCCTTTTCCTT) was used as housekeeping gene.

### Statistical analysis

Data are presented as mean ± 95% confidence interval (95% CI). The Mann–Whitney test was used for the immunofluorescence analysis for two‐sample comparisons. For real‐time PCR, the ΔΔC_*t*_ equation was used to determine relative expression, and the *P*‐value was calculated by two‐sided paired Student's *t*‐test based on the ΔC_*t*_ value.

## Results

### Tamoxifen interfered with pancreatitis‐induced extracellular matrix deposition in a sex‐specific manner

**Figure 1:**
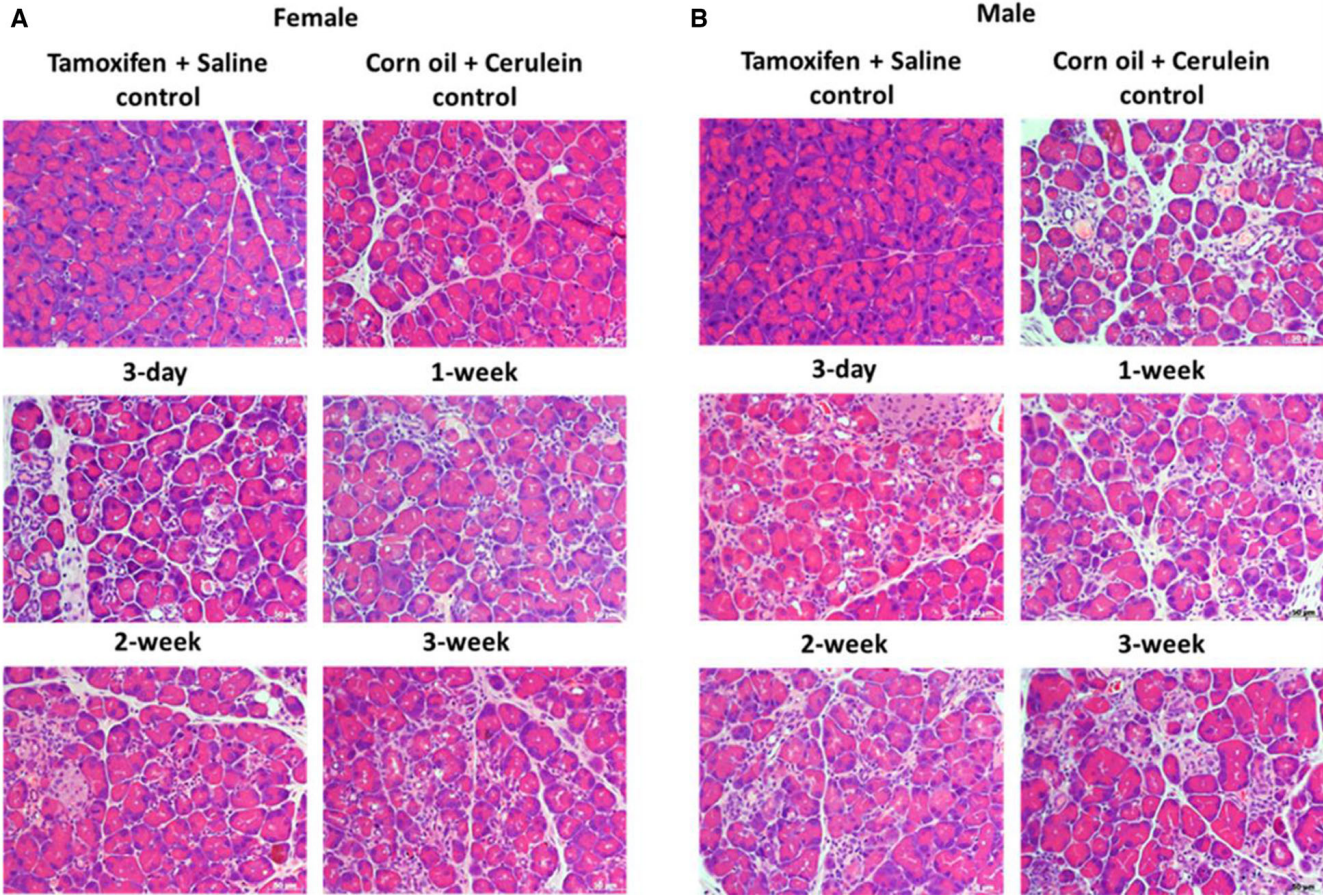
H&E staining of chronic pancreatitis tissue in each group. Normal acini are lost and replaced by progressive fibrosis associated with acinar‐to‐ductal metaplasia, and infiltration of fibroblasts and inflammatory cells. (A) Representative images of H&E staining of chronic pancreatitis tissue in female mice from each group. (B) Representative images of H&E staining of chronic pancreatitis tissue in male mice from each group. 200× magnification, scale bar: 50 μm.

We have previously demonstrated that the fibrotic response toward repeated cerulein stimulation is significantly increased in mice with a hypomorphic, general knockout for Smad7, a potent negative modulator of TGF‐β signaling 14. To gain further insight, we had planned to investigate the cell type‐specific role of Smad7 by using a conditional (‘floxed’) knockout allele of Smad7 17 under the control of *Col1a2‐Cre* ^*ERT*^ 12 in a C57BL/6J background. Results of a pilot experiment using only female control mice showed that mice treated with tamoxifen for 5 days plus cerulein after a 3‐day waiting period had a significantly higher fibrotic index than mice only treated with corn oil (vehicle for tamoxifen) plus cerulein (Fig. S1). In order to test if tamoxifen has a time‐specific and possibly also a sex‐specific influence, we induced chronic pancreatitis by a 2‐week cerulein treatment in female and male mice following different waiting periods after the last tamoxifen pretreatment (Fig. 1). Then, pancreata were evaluated by picrosirius red/fast green staining (fibrotic index) and immunofluorescence staining of collagen I, collagen IV, and fibronectin, the predominant biomarkers of fibrosis.

**Figure 2:**
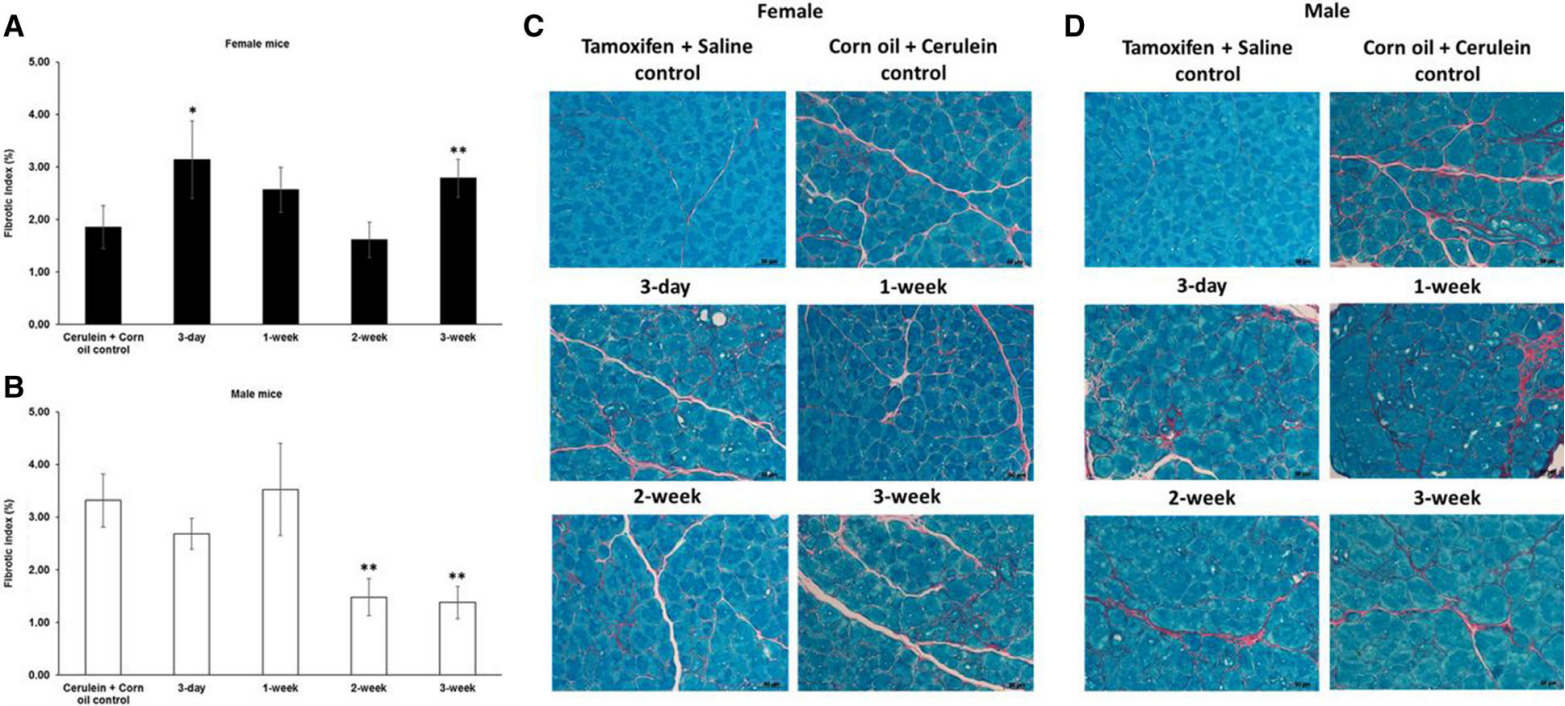
Quantification of the fibrotic index in chronic pancreatitis tissue. (A) Tamoxifen pretreatment followed by different washout periods significantly increased collagen‐based fibrotic index in female mice after 3‐day and 3‐week tamoxifen‐free periods. (B) Tamoxifen pretreatment decreased the expression in male mice after 2‐ and 3‐week tamoxifen‐free periods. The comparison was drawn to mice receiving only corn oil (vehicle) plus cerulein treatment commencing 3 days after the last tamoxifen gavage. (C, D) Representative images of picrosirius red/fast green staining of female and male pancreata at different time points. 200× magnification, scale bar: 50 μm. The values represent mean ± 95% CI (*n* = 5 for each group), (*) *P* < 0.05, (**) *P* < 0.01, Mann–Whitney test.

**Figure 3:**
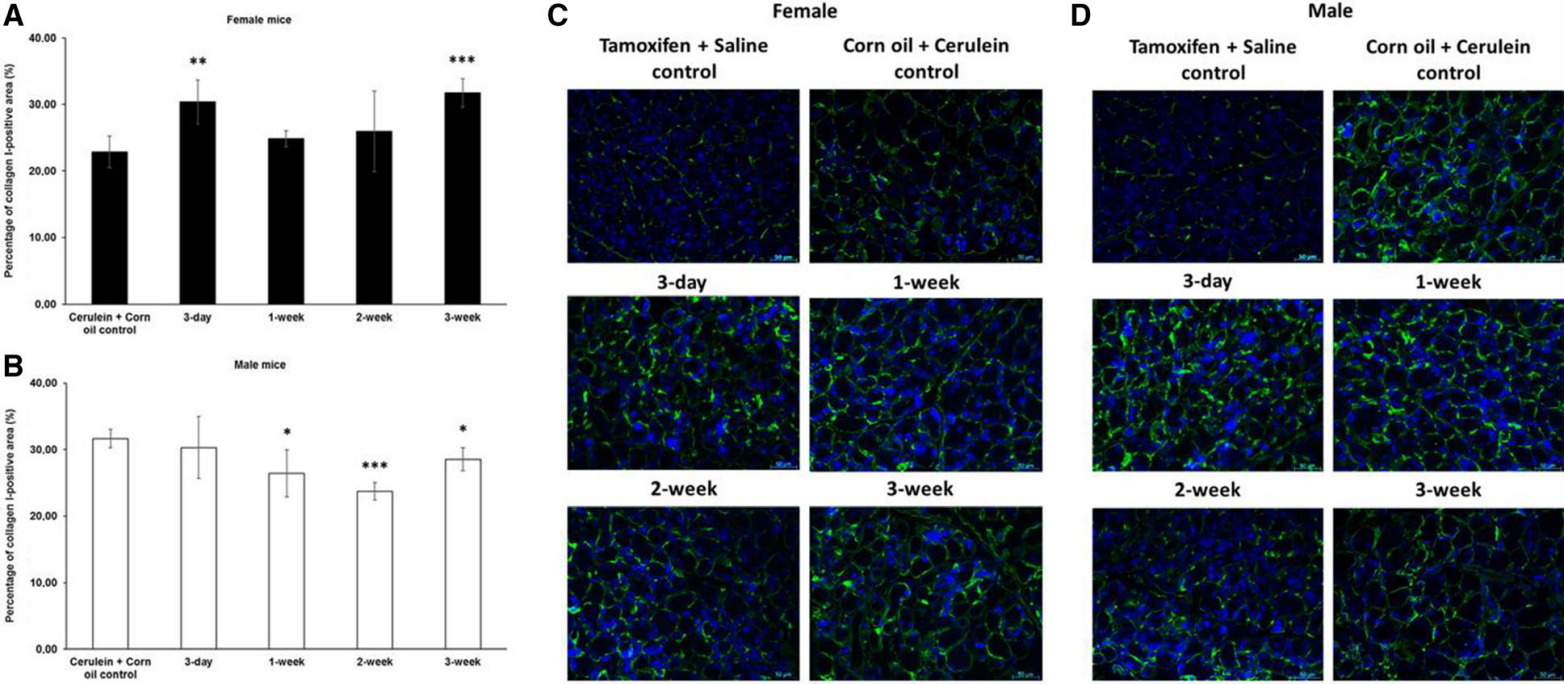
Immunofluorescence quantification of collagen I in chronic pancreatitis tissue. (A) Tamoxifen pretreatment followed by different washout periods significantly increased cerulein‐induced collagen I expression in female mice after 3‐day and 3‐week tamoxifen‐free periods. (B) Tamoxifen pretreatment decreased the expression in male mice after 1‐, 2‐, and 3‐week tamoxifen‐free periods. The comparison was drawn to mice receiving only corn oil (vehicle) plus cerulein treatment commencing 3 days after the last tamoxifen gavage. (C, D) Representative images of immunofluorescence staining of female and male pancreata at different time points. 200× magnification, scale bar: 50 μm. The values represent mean ± 95% CI (*n* = 5 for each group), (*) *P* < 0.05, (**) *P* < 0.01, (***) *P* < 0.001, Mann–Whitney test.

Like in the pilot experiment, we observed a higher fibrotic index in female mice after a 3‐day waiting time before cerulein induction (Fig. 2A,C). This finding was corroborated by staining for collagen I (Fig. 3A,C). Upon longer waiting periods, such as 1 or 2 weeks, the fibrotic index as well as the expression level of collagen I returned roughly to that of the control group, which received only corn oil and cerulein injections (Fig. 3A,C). However, the effect of tamoxifen on collagen I seemed to be oscillating, which was indicated by another increase in expression if pancreatitis was induced 3 weeks after tamoxifen application. Interestingly, tamoxifen generally decreased the fibrotic index (Fig. 2B,D) and collagen I deposition in male mice (Fig. 3B,D).

**Figure 4:**
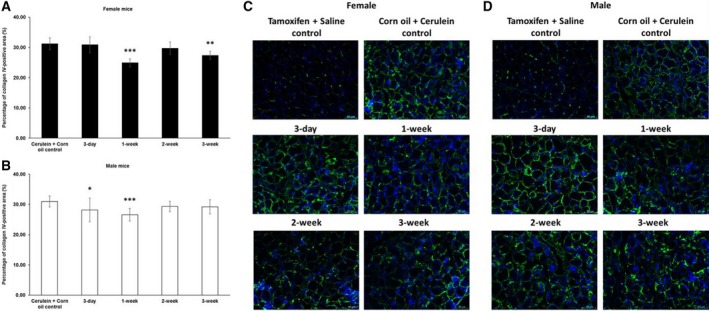
Immunofluorescence quantification of collagen IV in chronic pancreatitis tissue. (A) Tamoxifen pretreatment followed by different washout periods significantly decreased cerulein‐induced collagen IV expression in both female mice after 1‐ and 3‐week tamoxifen‐free periods and (B) male mice after 3‐day and 1‐week tamoxifen‐free periods. The comparison was drawn to mice receiving only corn oil (vehicle) plus cerulein treatment. (C, D) Representative images of immunofluorescence staining of female and male pancreata at different time points. 200 × magnification, scale bar: 50 μm. The values represent mean ± 95% CI (*n* = 5 for each group), (*) *P* < 0.05, (**) *P* < 0.01, (***) *P* < 0.001, Mann–Whitney test.

**Figure 5:**
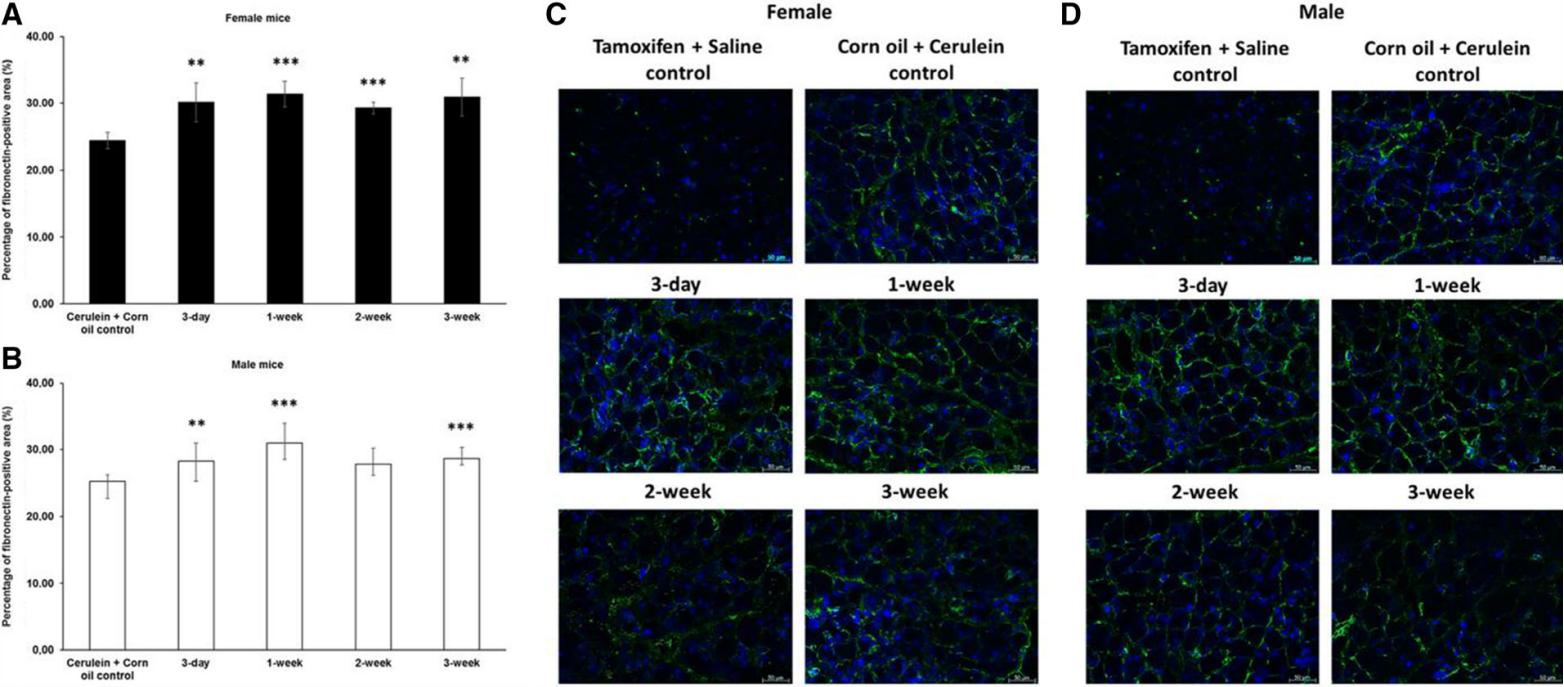
Immunofluorescence quantification of fibronectin in chronic pancreatitis tissue. Tamoxifen pretreatment followed by different washout periods significantly increased cerulein‐induced fibronectin expression in both female (A) and male (B) mice. The comparison was drawn to mice receiving only corn oil (vehicle) plus cerulein treatment. (C, D) Representative images of immunofluorescence staining of female and male pancreata at different time points. 200 × magnification, scale bar: 50 μm. The values represent mean ± 95% CI (*n* = 5 for each group), (**) *P* < 0.01, (***) *P* < 0.001, Mann–Whitney test.

In addition to collagen I, tamoxifen also affected collagen IV deposition. In female mice, we observed less collagen IV following a 1‐ or 3‐week waiting period. In male mice, we also found less collagen IV, however, after slightly shorter 3‐day or 1‐week waiting period (Fig. 4). For fibronectin, we observed yet another response to tamoxifen, namely a generally increased deposition of fibronectin in both female and male mice (Fig. 5).

### Tamoxifen treatment preceding pancreatitis induction had different effects on the number of fibroblasts and inflammatory cells in the pancreas of male and female mice

**Figure 6:**
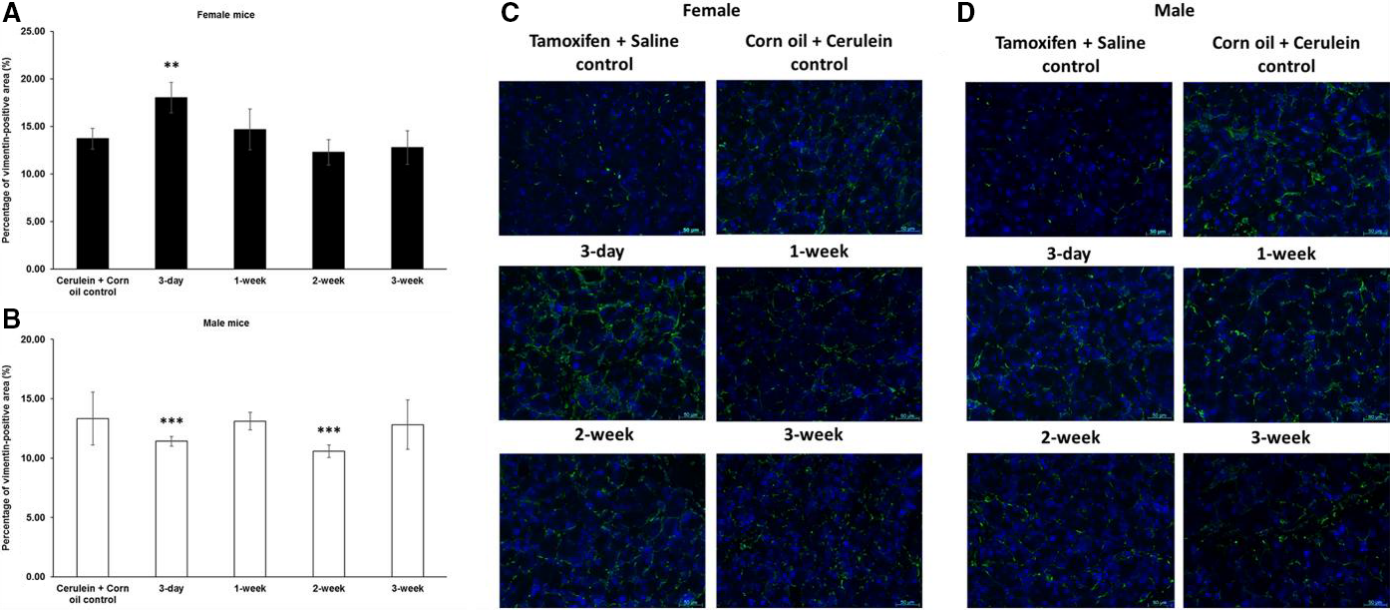
Relative quantification of PSCs by immunofluorescence of vimentin in chronic pancreatitis tissue. (A) Tamoxifen pretreatment followed by different washout periods significantly increased cerulein‐induced vimentin expression in female mice after a 3‐day tamoxifen‐free period. (B) Tamoxifen pretreatment decreased the percentage in male mice after 3‐day and 2‐week tamoxifen‐free periods. The comparison was drawn to mice receiving only corn oil (vehicle) plus cerulein treatment. (C, D) Representative images of immunofluorescence staining in female and male mice at different time points. 200× magnification, scale bar: 50 μm. The values represent mean ± 95% CI (*n* = 5 for each group), (**) *P* < 0.01, (***) *P* < 0.001, Mann–Whitney test.

Increased numbers of both myofibroblasts and inflammatory cells are characteristic for chronic pancreatitis. In order to investigate whether tamoxifen also had an effect on these two cell types, immunofluorescence staining of vimentin and F4/80, representative for PSCs/myofibroblasts and macrophages, respectively, was performed. Similar to the collagen I expression levels, pancreata from female mice presented with a higher number of PSCs/myofibroblasts when chronic pancreatitis induction was started only 3 days after tamoxifen pretreatment compared to vehicle pretreatment plus cerulein. This difference disappeared after longer waiting periods. However, the pancreata of male mice had generally lower numbers of PSCs/myofibroblasts when pretreated with tamoxifen compared to vehicle (Fig. 6).

**Figure 7:**
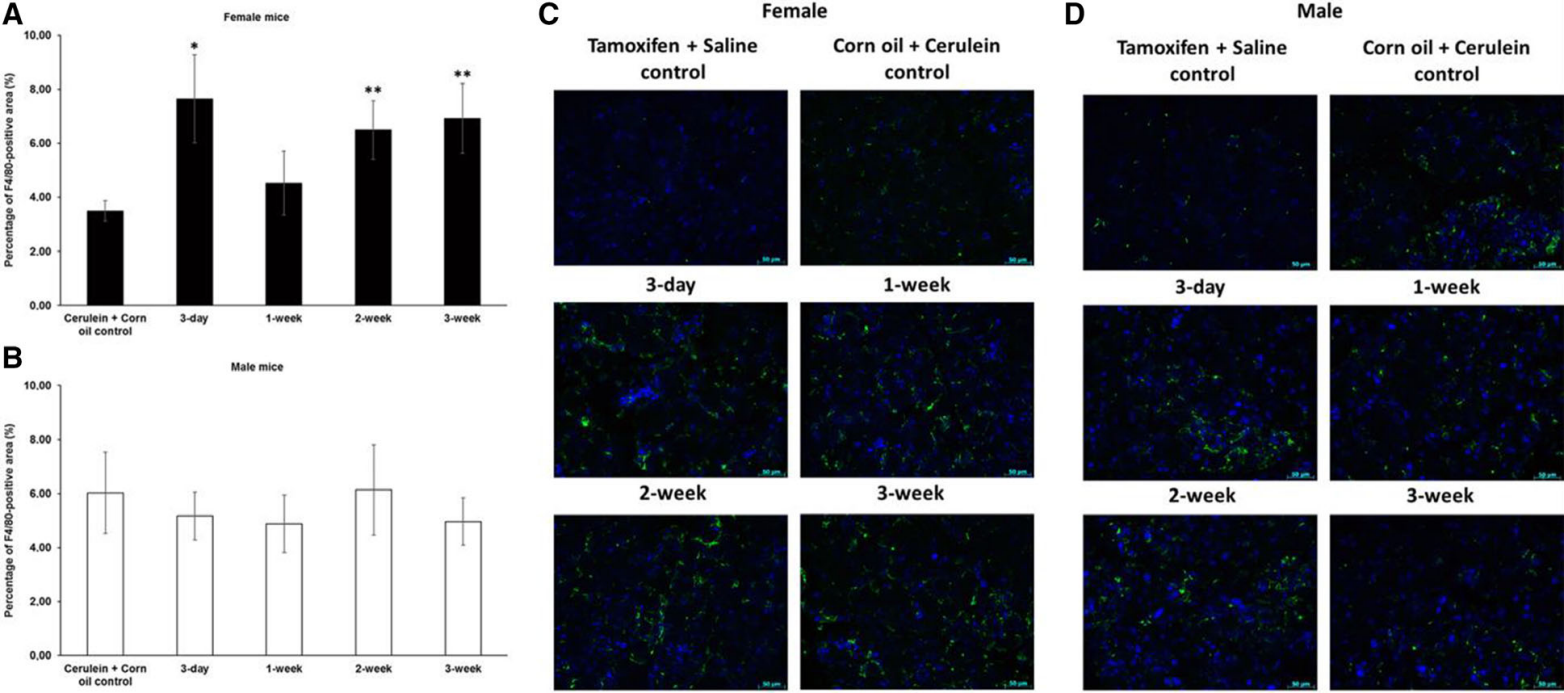
Relative quantification of macrophages by immunofluorescence of F4/80 in chronic pancreatitis tissue. (A) Tamoxifen pretreatment followed by different washout periods significantly increased cerulein‐induced F4/80 expression in female mice after 3‐day, 2‐week, and 3‐week tamoxifen‐free periods. (B) Trend of less F4/80 expression was observed after 3‐day, 1‐week, and 3‐week tamoxifen‐free periods in male mice. The comparison was drawn to mice receiving only corn oil (vehicle) plus cerulein treatment. (C, D) Representative images of immunofluorescence staining in female and male mice at different time points. 200× magnification, scale bar: 50 μm. The values represent mean ± 95% CI (*n* = 5 for each group), (*) *P* < 0.05, (**) *P* < 0.01, Mann–Whitney test.

Moreover, tamoxifen pretreatment resulted in mostly increased numbers of inflammatory cells in female mice, especially following the 3‐day, 2‐week, and 3‐week waiting periods. In male mice, on the other hand, we observed a trend of less inflammatory cells after a 3‐day, 1‐week, or 3‐week waiting time, although no statistically significant difference was observed (Fig. 7). Similar results were observed by CD11b staining, which in addition to macrophages also detects other granulocytes (data not shown). The oral gavage of tamoxifen alone without any cerulein treatment (saline controls) did not result in any signs of inflammation, fibroblast proliferation, or fibrosis.

### The effect of tamoxifen on pancreatic fibrogenesis might relate to estrogen receptor (ER) expression

TGF‐β has long been considered as the central mediator of fibrotic disease, causing myofibroblast differentiation and ECM deposition 18, 19. Recently, ER signaling has been reported to interfere with collagen synthesis 20. Tamoxifen, as a selective estrogen receptor modulator (SERM), has been shown to drive biological responses, which can be either estrogenic or anti‐estrogenic, depending upon the tissue in which its action was examined 21. In order to study the tamoxifen‐related mechanisms in modulating pancreatic fibrogenesis, we investigated whether cotreatment with tamoxifen and cerulein in primary mouse PSCs would reflect the differences observed in our *in vivo* experiments, and whether TGF‐β and/or ER signaling were involved.

**Figure 8:**
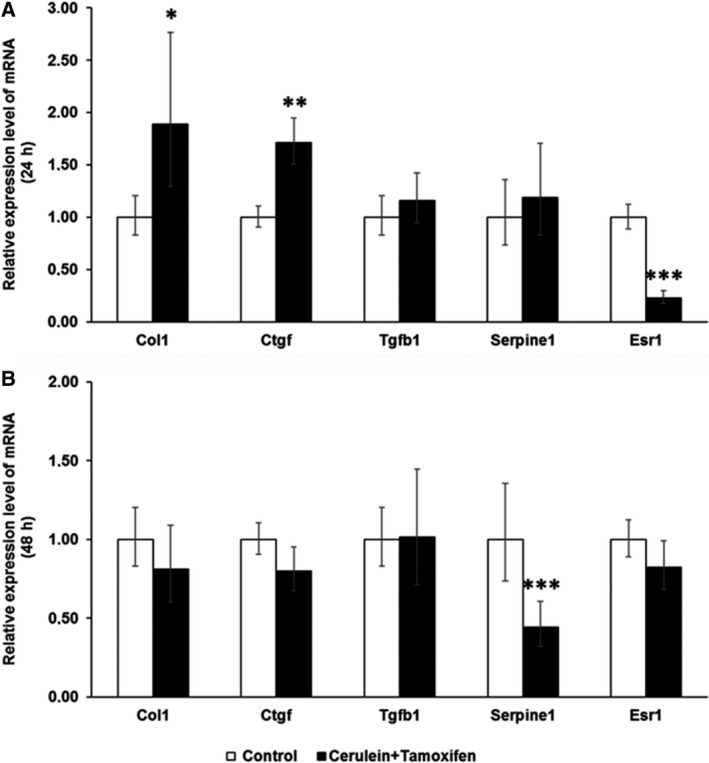
The mRNA expression level of *Col1a1, Ctgf, Tgfb1, Serpine1,* and *Esr1* was evaluated by RT‐PCR in female primary PSCs. (A) Female PSCs showed a significant upregulation of *Col1a1* and *Ctgf,* but a strong downregulation of *Esr1* expression level after 24‐h co‐incubation with 4‐hydroxytamoxifen and cerulein. (B) Female PSCs showed a significant downregulation of *Serpine1* mRNA expression after 48‐h co‐incubation with 4‐hydroxytamoxifen and cerulein. The relative expression levels were normalized to female PSCs treated only with cerulein plus tamoxifen vehicle (=1.0 arbitrary units) at the time points of 24 and 48 h, respectively. The values represent mean ± 95% CI (*n* = 5 for each group), (*) *P* < 0.05, (**) *P* < 0.01, (***) *P* < 0.001, Student's test.

Collagen I (*Col1a*1) mRNA, coding for the main component of ECM in fibrosis, was significantly upregulated after 24‐h cotreatment with tamoxifen and cerulein in female PSCs, compared with vehicle plus cerulein treatment (Fig. 8A). In line with this, the level of *Ctgf* mRNA, another classical TGF‐β target gene, was also higher in the tamoxifen cotreatment group. Surprisingly, *Tgfb1* and plasminogen activator inhibitor‐1 (*PAI‐1/Serpine1*) mRNA levels were not changed in female PSCs by tamoxifen cotreatment at the 24‐h time point. On the other hand, the ERα (*Esr1*) mRNA expression level was dramatically reduced (Fig. 8A). After 48‐h cotreatment, there was no more difference for these parameters with the exception of *PAI‐1/Serpine1*, which was downregulated (Fig. 8B).

**Figure 9:**
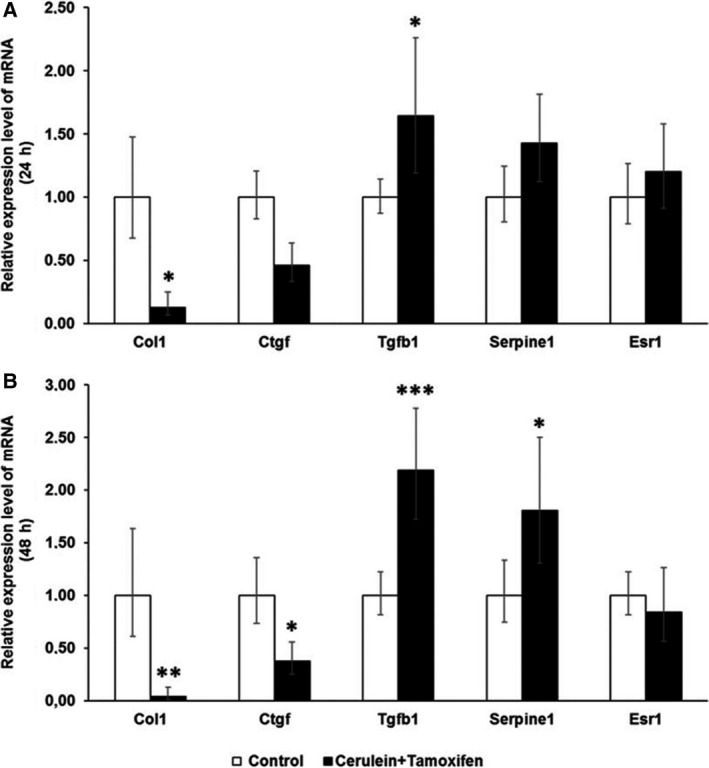
The mRNA expression level of *Col1a1, Ctgf, Tgfb1, Serpine1,* and *Esr1* was evaluated by RT‐PCR in male primary PSCs. (A) Male PSCs showed a significant downregulation of *Col1a1* but upregulation of *Tgfb1* expression level after 24‐h co‐incubation with 4‐hydroxytamoxifen and cerulein. (B) Male PSCs displayed a significant downregulation of *Col1a1* and *Ctgf* but upregulation of *Tgfb1* and *Serpine1* expression after 48‐h co‐incubation with 4‐hydroxytamoxifen and cerulein. The relative expression level was normalized to male PSCs treated only with cerulein plus 95% EtOH (=1.0 arbitrary units) at the time points of 24 and 48 h, respectively. The values represent mean ± 95% CI (*n* = 5 for each group), (*) *P* < 0.05, (**) *P* < 0.01, (***) *P* < 0.001, Student's test.

In male fibroblasts, *Col1a*1 mRNA was significantly reduced after 24‐ and 48‐h co‐incubation, going in hand with reduced *Ctgf* mRNA expression. Interestingly, there was an upregulation in both *Tgfb1* and *PAI‐1/Serpine1* whereas *Esr1* expression levels were unchanged (Fig. 9).

## Discussion

Tamoxifen‐inducible Cre‐LoxP systems have been widely used for the induction of genomic recombination in mice to provide important biological insights in tissue development, maintenance, and function in different disease models 2. The efficiency of Cre recombinases is affected by different factors, such as the age and strain of mice, the promoter driving the expression of the Cre recombinase, and the accessibility of LoxP sites in the respective target gene 22, 23. In addition to the above‐mentioned Cre‐related parameters, the activation of the CreERT system is furthermore affected by the pharmacokinetics of tamoxifen in relation to the different administration schemes of tamoxifen, which may vary in dosage, route, and multiplicity 3, 4, 23, 24. On the other hand, tamoxifen is not just an inducer for Cre^ERT^ recombinase, but has often neglected potential ‘off‐target’ effects in different tissues, which might be related to ER expression, as tamoxifen is a SERM eliciting tissue‐specific agonistic and antagonistic biological responses 5, 21, 25, 26. Recent studies have shown that tamoxifen can inhibit bone resorption and thus influence bone homeostasis 27. Exposure of a single dose of tamoxifen in pubertal mice is able to induce adverse testicular effects 24. Moreover, the utilization of tamoxifen in inducible Cre^ERT^‐LoxP systems seems to have long‐term side effects, which might confound the interpretation of time‐sensitive studies. For instance, tamoxifen can strongly attenuate CCl4‐induced hepatotoxicity in male C57BL/6N mice, even after a 10‐day tamoxifen exposure‐free period 4. It also interferes with fibrogenesis in obstructive nephropathy 5 and impairs the induction of pancreatic β‐cell proliferation during embryonic development 7. Therefore, the implementation of an optimized and adjusted tamoxifen treatment into the overall experimental scheme is required to achieve an efficient Cre recombination while minimizing ‘off‐target’ effects.

Tamoxifen's long‐term effect might be related to tissue‐specific pharmacokinetics, as well as its effect on ERα protein stability 28. Tamoxifen plasma levels have been reported to be undetectable 2 and 4 weeks after the treatment in mice 5. Another study reported that there was residual tamoxifen in the pancreatic islets up to 4 weeks after subcutaneous injections of tamoxifen 29. These different kinetics might relate to the distribution and metabolism of the lipophilic substance tamoxifen in different tissues. In rats, the highest levels of tamoxifen and its metabolites were observed in the lung and liver, followed by fat, kidney, and uterus. In addition, adipose tissue contained high concentration of the parent tamoxifen molecule and low concentrations of metabolites thereof because of the low activity of metabolizing enzymes in fat tissue 30. Thus, adipose tissue might represent a preserving peripheral compartment, which could interfere with tamoxifen plasma/tissue levels during and after tamoxifen administration.

The effects of tamoxifen in other fibrotic diseases have also been reported. It can actually ameliorate renal fibrosis 26, 31 and peritoneal fibrosis 25, 32. A sex‐dependent effect of tamoxifen was reported on obstructive nephropathy‐induced fibrosis in mice. Tamoxifen reduced fibrosis, accompanied by a reduction in nuclear Esr1 positivity in female mice, in contrast to male mice 5. Our data indicate an opposite tamoxifen effect on cerulein‐induced fibrogenesis in pancreas with higher a fibrotic index and higher amounts of collagen I in female pancreata and reduced fibrotic index/amounts of collagen I in male pancreata compared to vehicle plus cerulein‐treated mice of the respective sex (Fig. 1). This observation is supported by increased *Col1a1* and *Ctgf* mRNA together with a reduction of *Esr1* mRNA expression in tamoxifen plus cerulein‐incubated female PSCs, while no changes in *Tgfb1* mRNA levels were observed (Fig. 8). In male PSCs, we measured strongly reduced levels of *Col1a1* mRNA, despite slightly increased *Tgfb1* mRNA levels following tamoxifen plus cerulein cotreatment, while *Esr1* mRNA levels were unaffected (Fig. 9). In some studies, the fibrotic modulation property of tamoxifen has been reported to act via the TGF‐β 31, 32, 33, 34 or ERK1/2 pathway 35, 36. However, the influence of tamoxifen on TGF‐β signaling is still under debate, as opposing effects have been observed in different species and cell types 35, 37. Tamoxifen/ERα can either inhibit TGF‐β signaling 26 or enhance it 38, while other studies demonstrated that it does at least not interfere with the canonical Smad signaling 39.

TGF‐β is a major signaling pathway acting on remodeling of the ECM (ECM). In our *in vitro* experiments, it seems that tamoxifen modulates the activity of classical TGF‐β response genes/promoters in opposite directions depending on the sex of the PSCs (see *Col1a1/Ctgf* versus *Serpine1* in Figs 8 and 9). Our observation of opposite directions of *Tgfb1* mRNA and *Col1a1* mRNA expression in male PSCs after tamoxifen treatment has been observed in fibroblasts in other studies as well 32, 40. One possible explanation might be that tamoxifen enhances the TGF‐β‐induced expression of the inhibitory Smad7 to exert suppression of certain TGF‐β response genes 32, or higher levels of matrix metalloproteinase are generated, tipping the balance toward ECM degradation 41. In our experiments, the increased *Col1a1* mRNA in tamoxifen‐treated female PSCs seemed not related to the *Tgfb1* mRNA levels, suggesting that other mechanisms were involved in this scenario. Moreover, the sex difference of the tamoxifen effect was observed for deposition of collagen I rather than collagen IV (Figs 3 and 4) and fibronectin (Fig. 5) in our study. This indicates that the role of tamoxifen in modulating fibrogenesis differs for different ECM genes and ER‐related signaling might be involved in this sex specificity.

ERs, including Erα (*Esr1*) and Erβ (*Esr2*), act as ligand‐dependent transcription factors which translocate into the nucleus after ligand binding, modulating gene expression in conjunction with coactivators or corepressors 42. ERs regulate a wide variety of physiological processes in both the reproductive tract and nonreproductive tissues 21. The role of ER in the regulation of collagen biosynthesis has been investigated by using female mice lacking either *Esr1* or *Esr2*, and collagen content was significantly increased in the skin of *Esr1* ^*−*/*−*^ female mice but decreased in *Esr2* ^*−*/*−*^ female mice 20. Moreover, ESR1 expression was inversely correlated with liver fibrosis among patients infected with chronic HCV genotype 1b 43. The diverse impact of ESR1 and ESR2 on cardiac fibrosis has also been reported. Specific activation of *Esr1* and *Esr2* prevented cardiac fibrosis development, while *Esr2* deletion increased cardiac fibrogenesis 44, 45, 46. Interestingly, endogenous *Esr2* acts differently in male and female hearts. While *Esr2* promotes fibrosis in males, it inhibits fibrosis in female hearts 44. In our *in vitro* experiments, the *Esr1* mRNA level was significantly downregulated after 24‐h tamoxifen plus cerulein treatment in female PSCs (Fig. 8A), accompanied by increased collagen I deposition. In male PSCs, the *Esr1* mRNA level was not affected and the collagen I expression was greatly decreased (Fig. 9). *Esr2* mRNA was not detectable in our PSC preparations (data not shown). Together, these observations indicate that the effect of ER signaling on the fibrogenic process is not alike in all cell types, and the nature and extent of that response are determined by the combination of different transcription factors and coactivators/corepressors with the ERs on a given promoter 42. The contribution of tamoxifen‐mediated ER signaling in fibrogenesis thus needs further investigation. One recurring problem, which makes it difficult to interpret and compare many studies on tamoxifen effects, is that the sex of the investigated animals/cell lines, with the exception of breast cancer cells, is often not described.

The different levels of detected ECM proteins and the number of PSCs and inflammatory cells are altogether indicating a sex‐specific fibrogenic response following tamoxifen pretreatment in our study. Tamoxifen has been demonstrated to inhibit the activation and proliferation of fibroblasts *in vivo* and *in vitro* 5, 31, 47, but the sex aspect in these studies is unknown. With respect to immune cells, tamoxifen can result in reduced macrophage infiltration and proinflammatory cytokine expression in male mice 26, impaired dendritic cell (DC) differentiation, and reduction in the immunostimulatory capacity of DCs (sex not specified) 48. On the other hand, increased recruitment of Kupffer cells and leukocytes to damaged regions of tamoxifen‐pretreated livers of male mice was accompanied by complex changes in the expression of cytokines and immune‐related genes 4. Proinflammatory cytokine production was found to be either enhanced or dampened by ER activity 49, 50, 51. The change in the number of PSCs (Fig. 6) and macrophages (Fig. 7) in our *in vivo* study seems to support the sex‐specific collagen I accumulation/fibrotic index following tamoxifen pretreatment.

In conclusion, we have found that tamoxifen interferes with the cerulein‐induced chronic pancreatitis even after extended waiting periods before starting pancreatitis induction. This modulation property is reflected in a sex‐dependent fashion for collagen I deposition and in the numbers of PSCs and inflammatory cells. *In vitro* studies with male and female PSCs confirmed these results and hinted to an involvement of ER signaling. Dramatically reduced *Esr1* mRNA in female PSCs following tamoxifen treatment *in vitro* was paralleled by increased *Col1a1* and *Ctgf* expression. *In vivo*, we observed in female mice increased collagen I expression/fibrotic index and collagen IV and fibronectin expression, as well as increased numbers of PSCs/myofibroblasts and macrophages. In male fibroblasts, the mRNA level of *Esr1* did not change following tamoxifen treatment, while *Col1a1* and *Ctgf* mRNA levels were reduced. This was reflected *in vivo* by a reduced collagen I expression/fibrotic index and reduced numbers of PSCs/myofibroblasts. On the other hand, collagen IV and fibronectin levels are comparably increased in both sexes by tamoxifen treatment.

The side effects of tamoxifen are multiple with respect to kinetics, different cell types, and sex. For female mice, we found that a 2‐week waiting period after initial tamoxifen treatment and before cerulein administration resulted in a fibrotic response close to vehicle/cerulein‐treated mice. In the same experimental setup, male mice showed a generally reduced fibrotic response. Therefore, the optimal tamoxifen administration schedule can be diverse in different disease models and mouse strains, and specific pilot studies need to be performed according to the specific research question.

## Conflict of interest

The authors declare no conflict of interest.

## Author contributions

RH conceived and supervised the study, XL and CC performed experiments, XL analyzed the data, IK provided *Smad7* ^*fl/fl*^ mouse strain, and XL and RH wrote the manuscript.

## Supporting information


**Fig. S1.** The fibrotic index in different treatment schedules. Female *Smad7* ^*fl/fl*^ mice between 2 and 3 months of age were treated with tamoxifen oral gavage once per day for five consecutive days as described in Materials and methods. 3 days after the last oral gavage mice were subjected to 4‐week cerulein injection or saline injection twice per week as described in Materials and methods, respectively. In the control groups (Saline, Cerulein), mice were administrated with corn oil instead of tamoxifen, followed by 4‐week cerulein injection or saline injection 3 days after the last corn oil treatment. Mice were sacrificed on the third day after the last saline or cerulein injection. The collagen‐based fibrotic index was comparable between the mice treated by saline intraperitoneal injection with or without 5‐day tamoxifen pre‐administration. On the contrary, mice treated by cerulein intraperitoneal injection plus tamoxifen pre‐administration showed significantly higher fibrotic index than the group receiving only cerulein treatment. The fibrotic index was based on picrosirius red (HistoLab, Cat. No. HL27150.0500)/fast green counterstaining (Certistain^®^, Merck, Cat. No. 1.04022) by using paraformaldehyde‐fixed, paraffin‐embedded pancreatic tissue sections (4 μm). Five fields per section were selected randomly at 200× magnification and at least five sections per group were analyzed. The fibrotic index from picrosirius red /fast green staining was calculated as the percentage of collagen area in the total tissue area using the imagej software [1]. The values represent mean ± 95% CI (*n* = 8 for each group), (**)*P* < 0.01, Mann–Whitney test. [1] Schneider, C. A., Rasband, W. S. & Eliceiri, K. W. (2012) NIH Image to ImageJ: 25 years of image analysis, *Nature methods*. **9**, 671‐5.
