# Supplementary figures and images for: Tamoxifen affects chronic pancreatitis‐related fibrogenesis in an experimental mouse model: an effect beyond Cre recombination
Source: FEBS Open Bio. 2019 Sep 7;9(10):1756–68. doi: 10.1002/2211-5463.12714 (PMC6768287; doi:10.1002/2211-5463.12714)

**Supp fig 1**


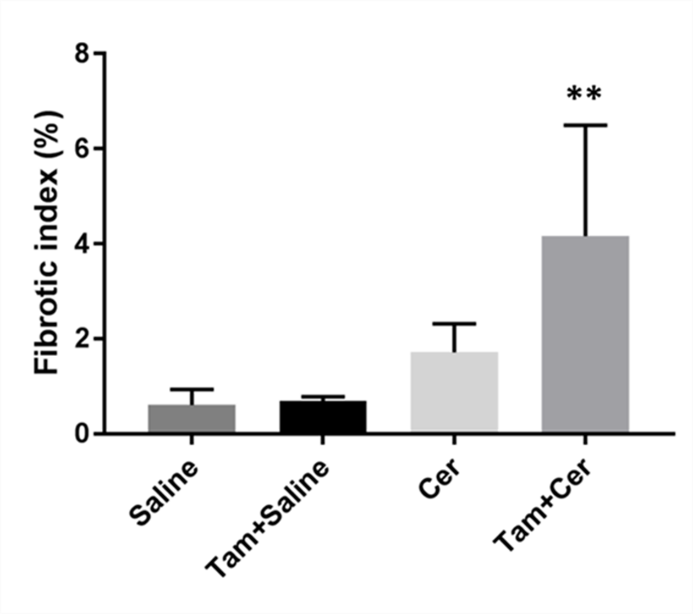

Supplement: Supplementary file 1 — Fig. S1. The fibrotic index in different treatment schedules. Female Smad7 fl/fl mice between 2 and 3 months of age were treated with tamoxifen oral gavage once per day for five consecutive days as described in Materials and methods. 3 days after the last oral gavage mice were subjected to 4‐week cerulein injection or saline injection twice per week as described in Materials and methods, respectively. In the control groups (Saline, Cerulein), mice were administrated with corn oil instead of tamoxifen, followed by 4‐week cerulein injection or saline injection 3 days after the last corn oil treatment. Mice were sacrificed on the third day after the last saline or cerulein injection. The collagen‐based fibrotic index was comparable between the mice treated by saline intraperitoneal injection with or without 5‐day tamoxifen pre‐administration. On the contrary, mice treated by cerulein intraperitoneal injection plus tamoxifen pre‐administration showed significantly higher fibrotic index than the group receiving only cerulein treatment. The fibrotic index was based on picrosirius red (HistoLab, Cat. No. HL27150.0500)/fast green counterstaining (Certistain®, Merck, Cat. No. 1.04022) by using paraformaldehyde‐fixed, paraffin‐embedded pancreatic tissue sections (4 μm). Five fields per section were selected randomly at 200× magnification and at least five sections per group were analyzed. The fibrotic index from picrosirius red /fast green staining was calculated as the percentage of collagen area in the total tissue area using the imagej software [1]. The values represent mean ± 95% CI (n = 8 for each group), (**)P < 0.01, Mann–Whitney test. [1] Schneider, C. A., Rasband, W. S. & Eliceiri, K. W. (2012) NIH Image to ImageJ: 25 years of image analysis, Nature methods. 9, 671‐5. [file FEB4-9-1756-s001.docx]
